# Antibody-Based Protective Immunity against Helminth Infections: Antibody Phage Display Derived Antibodies against B*m*R1 Antigen

**DOI:** 10.3390/ijms18112376

**Published:** 2017-11-22

**Authors:** Anizah Rahumatullah, Izzati Zahidah Abdul Karim, Rahmah Noordin, Theam Soon Lim

**Affiliations:** 1 Institute for Research in Molecular Medicine (INFORMM), Universiti Sains Malaysia, Minden 11800, Penang, Malaysia; anirah82@gmail.com (A.R.); ezzati87@gmail.com (I.Z.A.K.); rahmah8485@gmail.com (R.N.); 2 Analytical Biochemistry Research Centre, Universiti Sains Malaysia, Minden 11800, Penang, Malaysia

**Keywords:** single chain fragment variable (scFv), monoclonal antibodies, B*m*R1, phage display, immune library, naïve library

## Abstract

Helminth parasite infections are significantly impacting global health, with more than two billion infections worldwide with a high morbidity rate. The complex life cycle of the nematodes has made host immune response studies against these parasites extremely difficult. In this study, we utilized two phage antibody libraries; the immune and naïve library were used to identify single chain fragment variable (scFv) clones against a specific filarial antigen (B*m*R1). The V-gene analysis of isolated scFv clones will help shed light on preferential VDJ gene segment usage against the filarial B*m*R1 antigen in healthy and infected states. The immune library showed the usage of both lambda and kappa light chains. However, the naïve library showed preferential use of the lambda family with different amino acid distributions. The binding characteristics of the scFv clones identified from this work were analyzed by immunoassay and immunoaffinity pull down of B*m*R1. The work highlights the antibody gene usage pattern of a naïve and immune antibody library against the same antigen as well as the robust nature of the enriched antibodies for downstream applications.

## 1. Introduction

Human lymphatic filariasis infection remains a major public health problem, mainly in tropical countries. The World Health Organization (WHO) has identified lymphatic filariasis as the second leading cause of permanent and long-term disability in the world, after leprosy. It is caused by microscopic, thread-like worms, namely *Wuchereria bancrofti (W. bancrofti)*, *Brugia malayi (B. malayi)*, and *Brugia timori (*B. timori)** [1] The complex life cycle of human filarial nematodes, especially *W. bancrofti* and *B. malayi*, involves mosquitos as transmission vectors [2]. The complex life cycle of the nematodes is mainly attributed to the complicated host immune response, a direct reflection of the host–parasite interaction. Antibody responses against parasitic infections are a form of protection afforded by the human body. Immunoglobulin (Ig) E antibodies are mainly elevated in filarial infections but most are non-antigen induced responses. In addition to IgE, other antibody isotypes that are mainly regulated in response to filarial infections are IgG1 and IgG4 antibodies [3]. Studies with mice models have shown the role of IgE as well as IgM in conferring protection against *B. malayi* infections. There is also evidence of IgG acting as a potent mediator for antibody-mediated passive immunity in naïve animal models using purified IgG [4]. Therefore, it is likely that antibody repertoires from IgM and IgG could be potentially useful repertoires for the isolation of antigen-specific antibodies.

The flexibility of antibodies to counteract different parasites is evidenced by the exhaustive variability of antibody genes found in the body. This is the result of various gene-editing processes like VDJ gene rearrangements and somatic hypermutation that increases the diversity of the mature antibody sequences [5]. In order to study the different antibody gene usage patterns of a healthy and parasite-infected population, the IgM and IgG repertoires would be ideal for use. However, the IgG and IgM repertoire has to be replicated in vitro in order to investigate the differences experimentally. This can be achieved by using phage display technology where the repertoire is captured in a collection of cloned antibody sequences as a fusion to phage coat proteins to produce a diverse antibody library for panning [6]. A naïve antibody library will be representative of a healthy repertoire where no clinical symptoms of the disease are evident [7]. However, this does not discount the fact that sample collection in areas with high incidences could have been exposed to the parasite and recovered prior to collection. Therefore, memory responses against the exposed antigens would likely still persist in the repertoire, making a truly naïve repertoire impossible to achieve. Immune libraries, however, would focus on the repertoire from individuals that are infected and show clinical symptoms [7], providing primed antibody responses at the time of sample collection. Therefore, the naïve repertoire would be well defined by the IgM isotype, whereas the immune repertoire would be reflected by the IgG repertoire.

As the diversity of the antibody repertoire is dependent on VDJ recombination, a number of studies have been conducted to study the diversity of VDJ segments in response to various infections, malignancies, and autoimmune diseases [8,9,10,11,12]. Even with the increased interest in antibody gene usage, the focus on parasitic infections is scarce. In this study, an immune library for lymphatic filariasis (IgG isotype) [13] and a naïve library (IgM isotype) [14] was used to compare the ability of each library repertoire to generate monoclonal scFv clones against a specific filarial target. The target antigen used in this study is B*m*R1, a specific antigen used for the diagnosis of lymphatic filariasis. It is used as one of the target antigens for the PanLF Rapid test kit that is being used by the Malaysian Ministry of Health in the lymphatic filariasis elimination program. The B*m*R1 antigen is a good target as the model protein due to its specificity and sensitivity in the diagnosis of lymphatic filariasis. The antigen is expressed by the gene pPROEXHT/*Bm17DIII* (GenBank accession No. AF225296) and is highly specific and sensitive for IgG4 assays to detect the infection of both *B. malayi* and *B. timori*. The antigen has been used in various diagnostic setups with promising sensitivity levels of 93–100%. The *Bm*R1 antigen is also highly specific (99–100%) in terms of reactivity with sera from non-filarial infections, making it an ideal candidate for this work. The comparative focus on a single antigen would allow for a more systematic comparison to distinguish the gene segment use and complementarity determining region (CDR) mutations of both repertoires.

The work allowed us to shed some light on antibody responses, in particular the VDJ gene rearrangement and usage against a particular parasitic antigen. The isolated clones were characterized based on their gene sequence diversity and binding functionality. The scFv clones were evaluated for their functionality to identify the B*m*R1 antigen. A sequence comparison between the antibodies from both libraries was also carried out to identify preferential gene usage patterns by each repertoire. In conclusion, the study provided us critical insight to the preferential VDJ gene arrangement as well as complementarity determining region (CDR) mutation and length distribution of the scFv clones against the B*m*R1 antigen. In addition, the antibodies developed have potential diagnostic applications, evident from the results of the binding studies.

## 2. Results

### 2.1. Biopanning and Polyclonal ELISA

**Figure 1:**
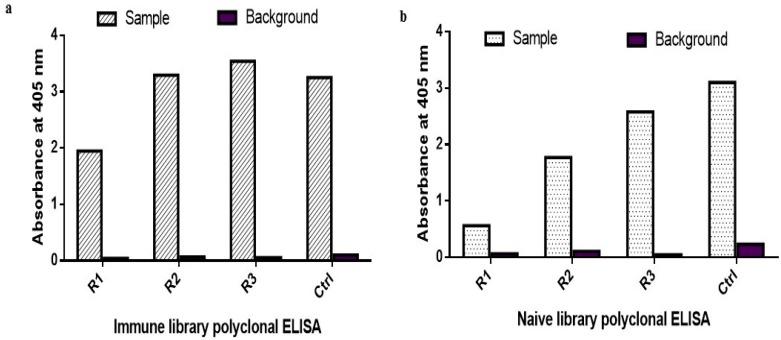
Polyclonal phage ELISA of B*m*R1 antigen during the panning cycles. (**a**) Immune library panning; (**b**) naive library panning. The positive control included was green fluorescence protein (egfp) to validate the assay. Bound phages were detected by incubation with HRP conjugated anti-M13 antibody (diluted 1:5000).

Phage particles displaying individual human scFv antibody fragments were selected by three rounds of biopanning on Maxisorp plates coated with B*m*R1 antigen using both naïve and immune libraries. The naïve library was previously developed using the repertoire from a pool of healthy donors. However, the immune library was developed using the repertoire obtained from individuals that were confirmed positive with filariasis infection. The observed increase in absorbance values indicates successful enrichment of target specific scFv presenting phage particles for each round of panning (Figure 1a,b). A significant increase in absorbance value for round 3 panning was obtained for both libraries. The positive and negative controls are indicative that the panning was successfully carried out with no background issues.

The starting amount of phage particles for the naive library was 1.32 × 10^12^ pfu and 3.85 × 10^10^ for the immune library. The amount of phage recovered from rounds 1 to 3 for the immune library was 4.15 × 10^7^, 2.65 × 10^5^, and 5.15 × 10^8^, respectively. Whereas, the amount of phage recovered from rounds 1 to 3 for the naïve library was 3.61 × 10^6^, 1.05 × 10^4^, and 6.21 × 10^7^, respectively.

The enrichment ratio for the immune library was 1.07 × 10^−3^, 0.69 × 10^−5^, and 1.33 × 10^−2^, respectively. The enrichment ratio for the naive library was 2.73 × 10^−6^, 7.9 × 10^−9^, and 4.7 × 10^−5^ for the three rounds, respectively. The enrichment for the immune library from round 2 to 3 compared to round 1 is 0.0064- and 12.42-fold. The enrichment for the naïve library is 0.0029- and 17.2-fold.

### 2.2. Monoclonal scFv ELISA

**Figure 2:**
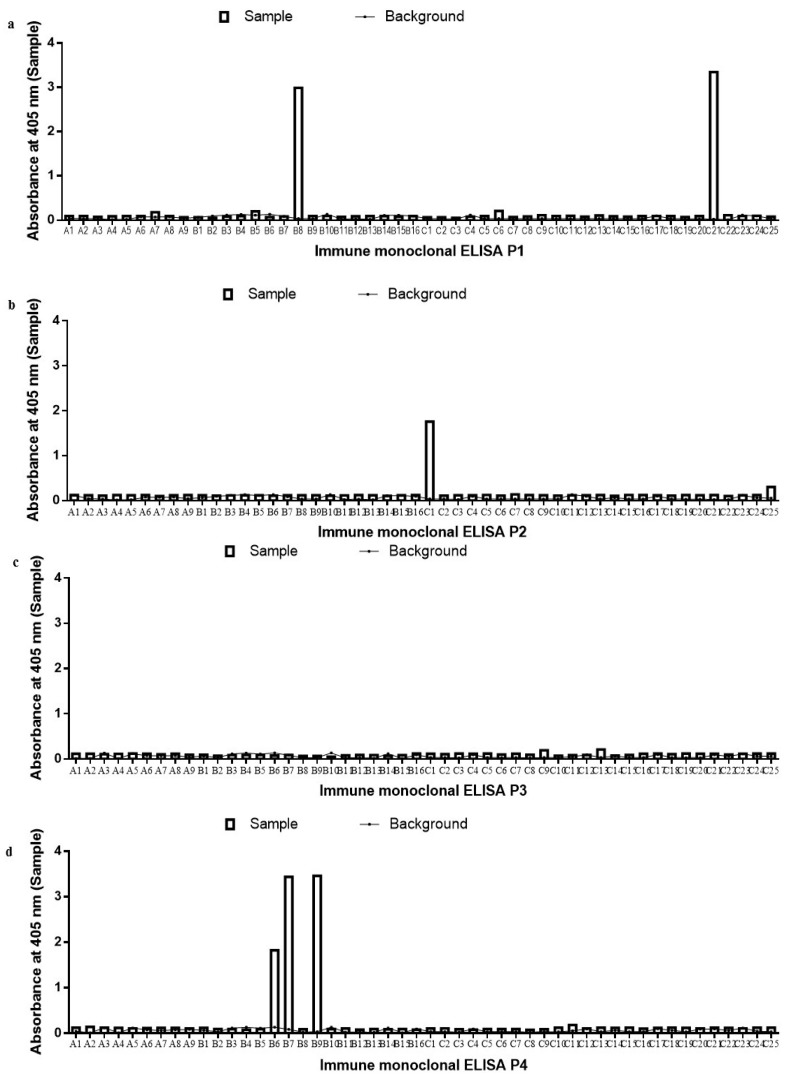
Monoclonal phage ELISA analysis of scFv clones from the immune library against *Bm*R1 antigen. Bound phages were detected by incubation with HRP conjugated anti-M13 antibody (diluted 1:5000). (**a**) Plate 1 of monoclonal phage ELISA, (**b**) Plate 2 monoclonal phage ELISA, (**c**) Plate 3 monoclonal phage ELISA, (**d**) Plate 4 monoclonal phage ELISA.

**Figure 3:**
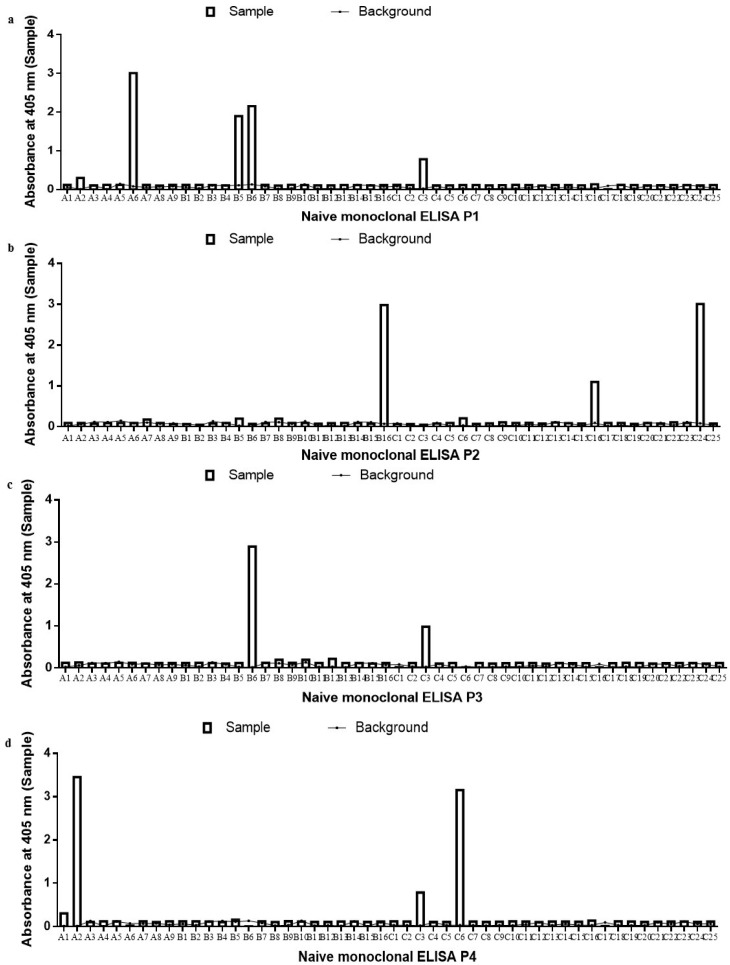
Monoclonal phage ELISA analysis of scFv clones from the naïve library against *Bm*R1 antigen. Bound phages were detected by incubation with HRP conjugated anti-M13 antibody (diluted 1:5000). (**a**) Plate 1 of monoclonal phage ELISA, (**b**) Plate 2 monoclonal phage ELISA, (**c**) Plate 3 monoclonal phage ELISA, (**d**) Plate 4 monoclonal phage ELISA.

A total of 392 scFv clones from both libraries were analyzed using monoclonal scFv phage ELISA. The monoclonal scFv phage ELISA resulted in six positive binders identified for the immune library and 11 positive binders for the naïve library (Figure 2a–d and Figure 3a–d). Positive clones were decided with a cut-off OD_405_ value above 0.5 after taking into consideration the background value. The range of the OD_405_ values of positive clones was 0.69 to 3.58. The immune and naïve library were able to provide satisfactory enrichment of B*m*R1-specific scFv clones. The overall background readings obtained from all the clones are relatively low (Figure 2a–d and Figure 3a–d).

### 2.3. Antibody Sequence and Gene Analysis

The positive scFv clones were then sequenced and analyzed using IMGT/VQUEST and VBASE2 to determine the human germline sequences of each clone. The sequence analysis showed that only four out of six scFv clones for the immune library and eight out of 11 naïve scFv clones exhibited complete gene sequences without any mutations. The remaining sequences for both libraries had mutations resulting either in a frame shift or truncation. The clones with problematic sequences were then omitted from further analysis.

**Figure 4:**
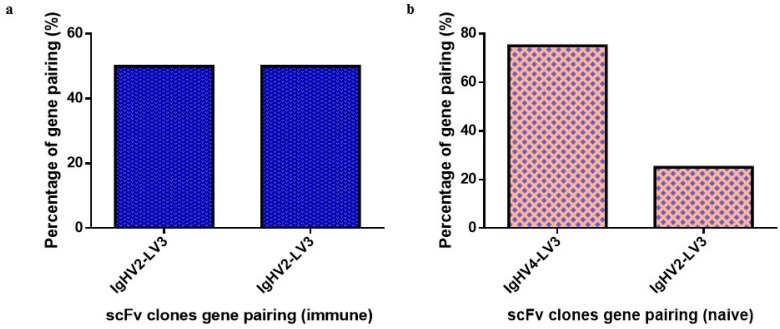
Analysis of gene pairing frequency of human scFv antibodies selected against B*m*R1 antigen. (**a**) Immune library; (**b**) naïve library. Figures are presented as a percentage of the total number of scFv sequenced.

The scFv clones from the immune library had an equal representation of IgHV2–Vλ3 (50%) and IgHV2–Vκ3 (50%) (Figure 4a). The scFv clones from the naïve library showed variations in gene distribution with two scFv sequence pairings being identified. Six out of eight clones were derived from the IgHV4–Vλ3 (75%) pairing and the other two sequences showed IgHV1–Vλ1 (25%) pairing (Figure 4b). The clones from the immune library showed a preference for the VH2 family only. However, clones from the naïve library showed no representation of the Vκ family, with a majority of clones utilizing the IgHV4–Vλ3 combination.

### 2.4. CDR Length Analysis

**Figure 5:**
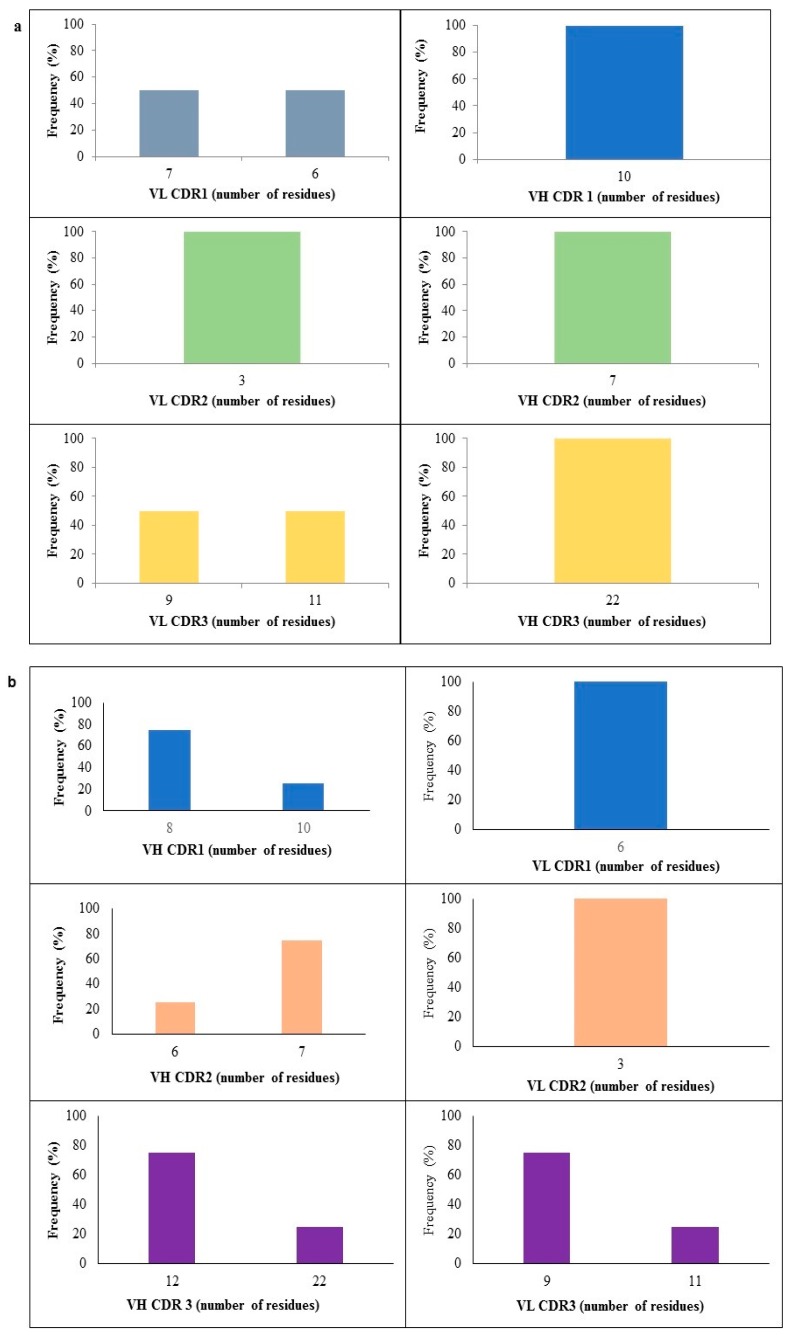
Amino acid length variations of IgHV and IgLV of B*m*R1 scFv clones. (**a**) Immune library; (**b**) naïve library. Length distribution of VH CDR1, CDR2, and CDR3 regions showing highly diversified CDR3 repertoires with different lengths.

The antigen-binding site of antibodies is usually formed by the combination of six loops of hypervariable sequences, three CDRs (CDR1, CDR2, and CDR3) from the HC and LC, respectively. In general, the CDR length for the VH is between seven and 22 amino acids. The HC CDR1 was found to be eight and 10 amino acids in length. CDR2 had only six and seven amino acids, whereas for CDR3 the distribution was 12 and 22 amino acids. The LC analysis showed six and seven amino acids for CDR1, only three amino acids for CDR2, and 9 and 11 amino acids for CDR3 (Figure 5a,b).

### 2.5. Amino Acid Distribution and Polarity Analysis

**Figure 6:**
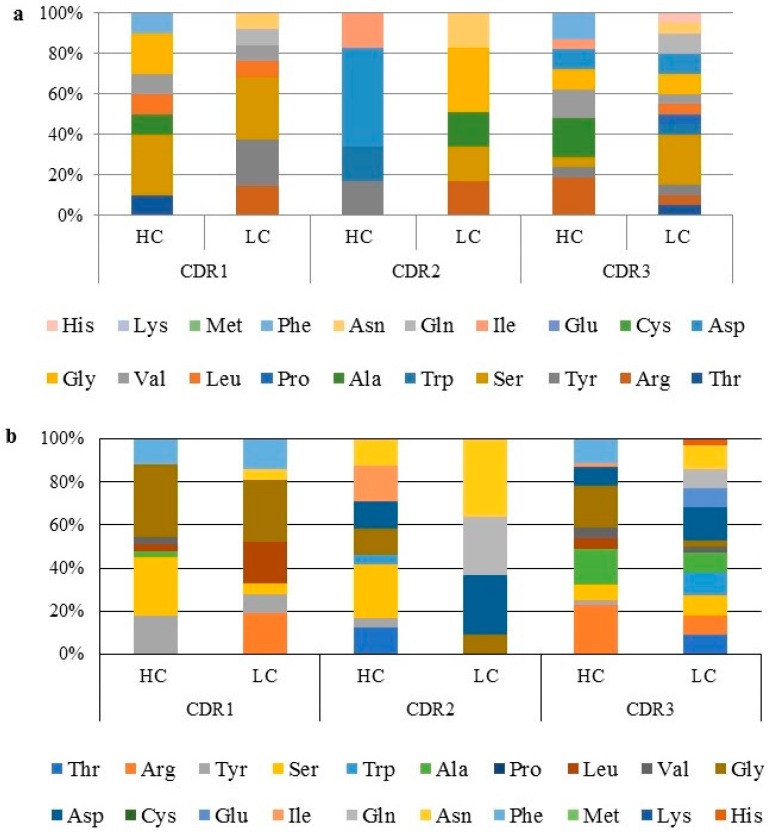

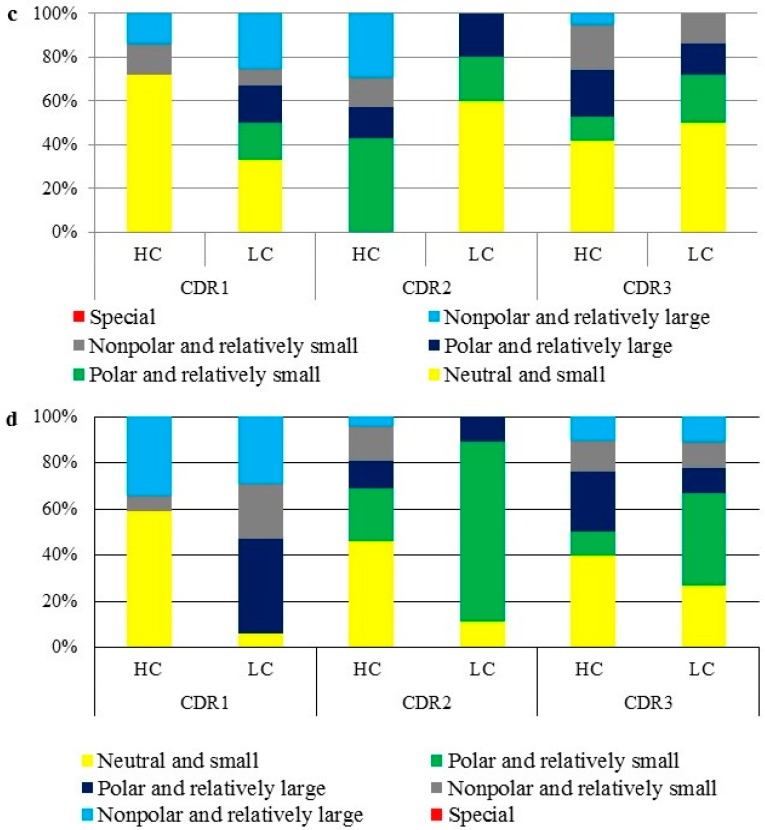
Analysis of amino acid distribution and polarity of anti-B*m*R1 scFv clones. CDR1, CDR2, and CDR3 showing variations in distribution of amino acids. (**a**) Amino acid distribution of immune library derived clones; (**b**) amino acid distribution of naïve library derived clones; (**c**) amino acid polarity of immune library derived clones; (**d**) amino acid polarity of naïve library derived clones.

The amino acid propensity of the enriched scFv clones showed a random distribution pattern for antibody clones obtained from both libraries (Figure 6a,b). The abundance of each individual amino acid for the HC and LC was assessed. Increase in the presence of different amino acids can be seen from CDR1 to CDR3. CDR3 showed the highest variation in amino acids compared to CDR1 and CDR2. The amino acid distribution pattern showed certain amino acids being significantly over represented, under represented or even absent in some cases. This variation highlights the diversity of the antibody repertoire within a small subset of clones against a particular antigen.

The CDR1 of the HC from the immune library clones showed a higher representation of serine (S), followed by glycine (G). However, CDR2 of the HC was predominantly aspartate (D), with arginine (R) and alanine (A) abundant in CDR3 of the HC. On the other hand, the CDR1 of the LC had a higher percentage of serine (S) and tyrosine (Y). Glycine (G) was highest in CDR2 and serine (S) was predominant for CDR3. Glutamate (E) was found to be absent from the antibody repertoire of the immune library clones. The HC CDR analysis of naïve library clones showed a high representation of G, followed by S in CDR1. However, isoleucine (I) was predominant in CDR2, whereas R and G were high in CDR3. LC CDR1 had a high representation of G, with asparagine (N) and D for CDR2 and CDR3, respectively. The clones from the immune and naïve library showed the absence of methionine (M), lysine (K), and cysteine (C). However, glutamate (E) and proline (P) were additionally missing from the immune and naïve repertoires, respectively.

Underrepresented amino acids were found to be histidine (H), valine (V), leucine (L), tryptophan (W), and threonine (T). The polarity distribution for all CDRs was rather similar, with a higher representation of neutral and small amino acids except for the CDR2 of the HC, where no representation of this group of amino acids were visible (Figure 6c,d). Absence of polar amino acids in CDR1 of the HC for both libraries was evident, whereas the CDR2 of the LC from the immune library showed the absence of non-polar amino acids.

### 2.6. Soluble Expression of scFv Clones

**Figure 7:**
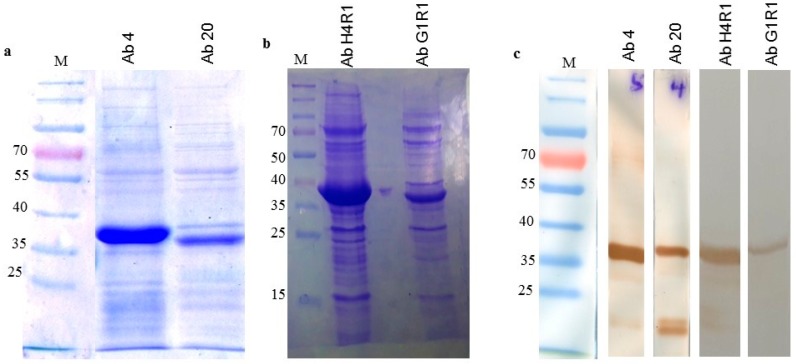
Soluble scFv protein analysis with SDS PAGE and Western blotting. (**a**) SDS PAGE of immune scFv proteins; (**b**) SDS PAGE of naïve scFv proteins; (**c**) Western blot analysis using anti-Cmyc secondary antibodies for all scFv proteins.

Binding characterization was performed using the unique clones from each gene family. In total, there were two unique scFv clones for both immune and naïve library. Clones Ab 4 and Ab 20 were identified from the immune antibody library whereas clones Ab H4R1 and Ab G1R1 were isolated from the naïve library. All the soluble antibodies from both libraries were successfully expressed and purified. Western blot analysis using anti-Cmyc was able to show the presence of a band at approximately 37 kDa that confirms the presence of the soluble scFv clones (Figure 7a,b).

### 2.7. Binding Characterization

**Figure 8:**
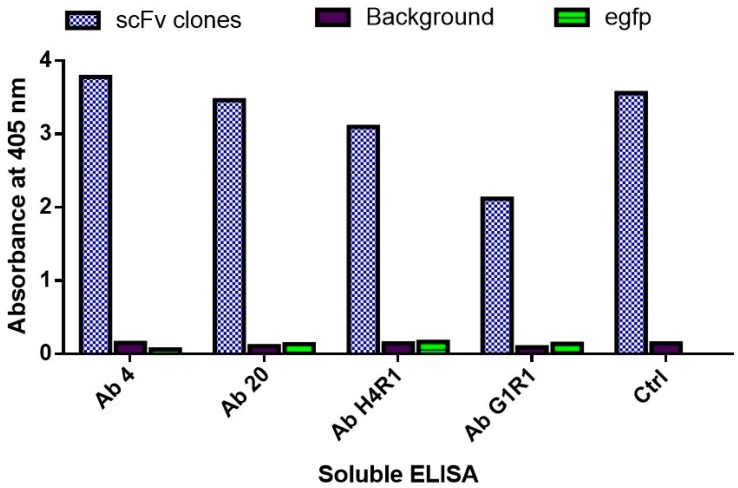
Immunoassay analysis of isolated scFv clones against B*m*R1 antigen with controls.

The soluble scFv clones were subjected to immunoassay analysis against the target antigen. This was done to confirm that the binding ability of the clones was maintained in soluble form. Figure 8 shows the OD readings of all four scFv clones when incubated with the B*m*R1 antigen. As the amount of scFv used for each sample was kept constant, a direct comparison shows that clone Ab G1R1 had the lowest OD readings. The OD readings of the control sample tested using an in-house anti-eGFP antibody showed good OD readings to confirm the assay was functioning well. The negative control samples did not show any OD readings, as expected. All four clones were tested against the positive control protein (eGFP) and did not show any cross-reactivity to the background and control protein.

**Figure 9:**
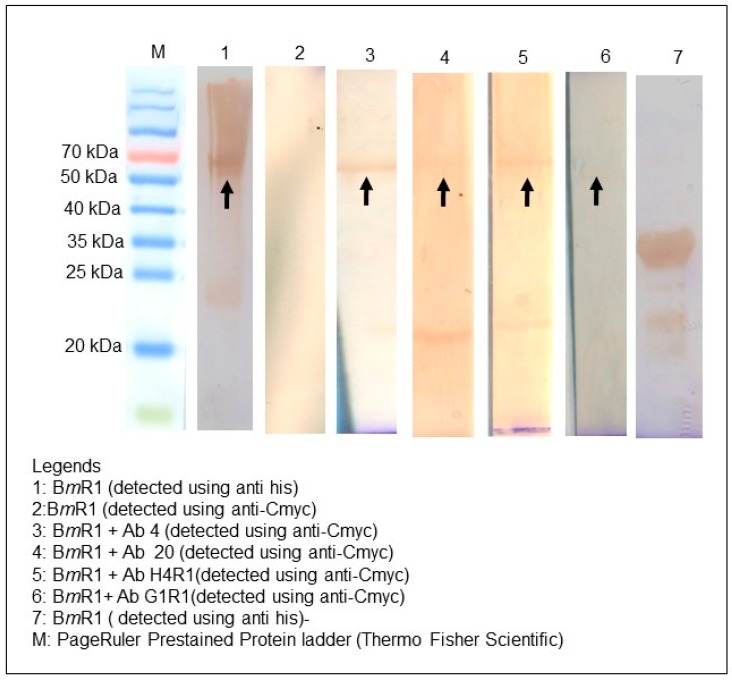
Western blot analysis of the identified scFv clones against B*m*R1. All the scFv clones were tested with the target antigen (B*m*R1) to confirm binding. Lane 1 to 6 Western blot was done using non-reducing SDS PAGE. Lane 7 shows the sample from a reducing SDS PAGE. The arrow shows the target protein (B*m*R1).

A non-reducing Western blot analysis was performed to identify potential binding of the scFv clones to a conformational epitope of the antigen (Figure 9). During non-reducing SDS PAGE, only the quaternary structure of the protein breaks but the disulfide bonds stay intact. Lane 1 shows the detection of the intact B*m*R1 antigen protein using anti-his secondary antibodies. Lane 2 highlights that the antigen protein does not react with anti-Cmyc secondary antibodies and Lane 7 shows the size of the antigen with anti-his secondary antibodies under reducing SDS PAGE. Lanes 3 to 6 show the binding of the scFv proteins to the antigen. A reducing scale of band intensity for the clones would start with clone Ab 4 as the highest, then clone Ab H4R1 followed by clone Ab 20 and lastly clone Ab G1R1.

### 2.8. Competitive Analysis of Identified scFv Clones

**Figure 10:**
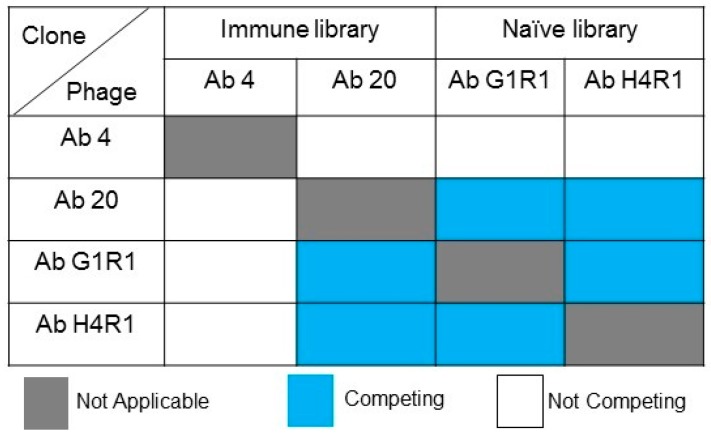
Summary of competitive ELISA of identified scFv clones.

The binding region of antibodies can potentially bind to similar epitopes, although the sequence of the paratope on the antibody may differ. Thus, competing a pair of scFv clones against the same target antigen could provide information on the epitope targeted by the individual scFv clones. The isolated scFv clones from the immune and naïve libraries competed against each other in a competitive ELISA to examine the binding characteristics of the clones. A pair of clones that competes to bind at the same binding site will result in low absorbance readings. Meanwhile, the non-competitive clones will result in a high absorbance reading, indicating interaction with different epitopes of the B*m*R1 antigen. All four scFv clones are represented by a set of unique gene families. The immune scFv clones Ab 4 and Ab 20 did not exhibit competition with each other, indicating that both clones are binding to different interaction sites on the target antigen. However, naïve scFv clones Ab H4R1 and Ab G1R1 exhibited direct competition for the same binding site with a low absorbance reading. In addition, these two scFv clones only cross-react with clone Ab 20 from the immune library (Figure 10).

### 2.9. Antibody Titration ELISA

**Figure 11:**
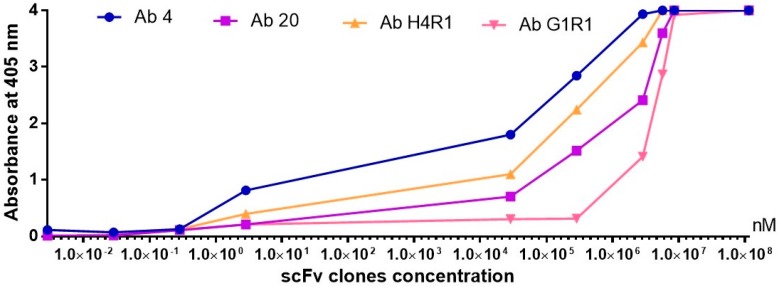
Result of antibody titration ELISA using immune and naïve library derived scFv clones. Detection using 1:2500 HRP conjugated anti C-myc.

The aim of a titration ELISA was to provide a ranking system for the identified scFv clones based on their binding properties. The range of antibody concentration used was 400 μg (1.143 × 10^8^ nM) to 100 fg (2.86 × 10^−3^ nM) per well. The titration ELISA was able to show the rank of the scFv clones based on the amount of antibodies required for antigen detection. The scFv clone with the best binding ability was clone Ab 4, which maintained binding at the lowest concentration of antigen of 10 pg (2.86 × 10^−1^ nM) (Figure 11). The ranking of the identified clones from the best to the worst performing was Ab 4, Ab H4R1, Ab 20, and Ab G1R1.

### 2.10. Immunoaffinity Pulldown Using Identified scFv

**Figure 12:**
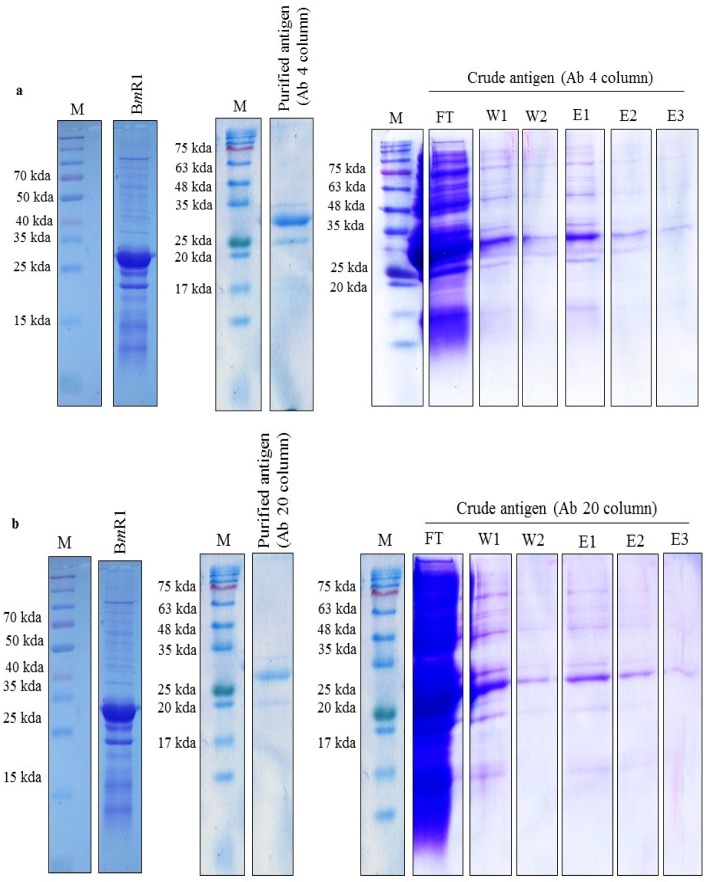
SDS PAGE analysis of B*m*R1 antigen immunoaffinity pulldown. (**a**) Evaluation of binding using purified and crude B*m*R1 antigen with Ab 4 column; (**b**) evaluation of binding using purified and crude B*m*R1 antigen with Ab 20 column. Lane FT: flow through; Lanes W1–W2: washing; Lanes E1–E3: elution.

The scFv clones Ab 4 and Ab 20 were selected for the immunoaffinity pulldown experiment based on the yield and purity of the soluble fraction. The scFv soluble proteins were successfully coupled and packaged to make an affinity column. The coupling efficiency was satisfactory with 80% for clone Ab 20 and 75% for clone Ab 4. The columns were evaluated with the capture of his-tag purified B*m*R1 and the specificity was good. Both columns were successfully applied to capture the target antigen in the crude fraction. However, clone Ab 4 showed a better target antigen recovery than clone Ab 20 in the SDS PAGE. The columns generated using both scFv clones were able to capture and release the target antigen specifically to yield a purified band of the target antigen (Figure 12a,b). This shows the ability of the clones to specifically capture and release the target antigen from a collection of non-random proteins.

## 3. Discussion

In vitro display and selection technologies like phage display are commonly used methods to generate recombinant human antibodies against a panel of target antigens for research, diagnostic, and therapeutic applications [15,16,17,18]. In general, there are three main types of antibody libraries, namely the naïve, synthetic, and immune antibody libraries [19]. The main difference between these libraries is the origin of the antibody repertoire used to construct the library [6]. Naïve library repertoires are mainly made up of naturally unskewed repertoires that provide an attractive source of repertoire diversity. Synthetic library repertoires are similar to naïve libraries in terms of diversity. However, the source of repertoire differs as the repertoire is generated by synthetic oligonucleotides that are typically randomized at defined positions in the CDR region of the antibody. On the other hand, immune libraries are generated from a specialized repertoire as the antibody genes are obtained from B-lymphocytes isolated from infected patients of a particular disease. This in turn results in a skewed preference of antibodies and a preferential gene usage. In terms of lymphatic filariasis, a direct comparison of antibodies against parasitic antigens like B*m*R1 has never been reported. A direct comparison would allow a better understanding of the antibody gene usage preference in correlation to the binding ability against a specific target antigen.

Generally, immune libraries are used to generate high-affinity antibodies against antigens of a specific disease due to the skewed antibody repertoires [20]. However, this may not be a feasible prospect for many researchers since new libraries need to be constructed for every different antigen. Therefore, naïve libraries are preferred instead of immune libraries for the isolation of antibodies against multiple antigens. Even so, the impact and benefits of immune libraries in antibody generation should not be neglected. This is because antibodies derived from immune libraries tend to present higher affinity clones in comparison to naïve libraries due to their skewed nature [21]. Therefore, a direct comparison to highlight the functionality and repertoire diversity of the clones derived from the immune libraries will offer a deeper understanding of the repertoire diversity of immune libraries.

The data show the ability of both immune and naïve libraries to generate scFv clones that bind to the B*m*R1 antigen. The visible variation is not restricted to the binding ability but is also at the gene level. The immune library showed an equal representation of gene usage from the lambda and kappa families. However, the naïve library only had gene usage from the lambda family, indicating a preference for lambda family by scFv clones derived from the naïve library for that particular antigen. Studies have shown that the dominating variable HCs for naïve libraries are VH1 and VH3 and in some instances VH4. For the light chain, it is mainly Vλ1, Vλ2, Vλ3 and in some cases Vλ6 [22,23]. This supports the representation of the heavy and light chain obtained in this study from the naïve library clones. In another study, a preference for the VH1, VH3, and VH4 gene families was seen in antibodies isolated from *Trypanosoma cruzi* infected patients using an immune library [24]. The study on *Entamoeba histolytica* antibody generation yielded a dominant VH3 gene usage [25]. However, this is in contrast with the V-gene preference for the clone derived from the filariasis immune library, which showed VH2 as the preferred V gene. It must be noted that variation in gene usage may also be a result of the different antigens targeted.

The amino acid distribution of clones from both libraries showed good randomization of the CDR regions for both VH and VL regions with higher levels of neutral and small amino acids. The presence of smaller amino acids in the CDR regions is beneficial for CDR loop flexibility, which may have a positive effect on antigen binding [26,27,28]. The variation in the CDR length directly contributes to the topographical variation of the binding sites for improved binding characteristics [29]. The most important and diverse region for the antibody-combining site is the CDR3 of the heavy chain [30,31,32]. Previous studies have addressed the importance of certain amino acids for higher affinity and specificity antibodies. The clones from this work showed an abundance of R, A, and G residues in CDR3 in comparison to other amino acid residues. A higher representation of R residues was expected because many studies on structural characteristics have reported the presence of large R residues at the active sites, which mediates a wide range of intermolecular interactions [33,34,35]. These residues are also preferred on the surface as they play an important role in protein stability with the formation of salt bridges [34,36]. Small residues such as G and A are also dominating residues that contribute to high-affinity antigen recognition [34]. This is because small amino acids are postulated to provide greater conformational flexibility and prevent steric crowding [30].

There were significant differences noticed in amino acid distribution patterns between scFv clones derived from the naïve and immune library. Certain amino acids were found to be higher in affinity-matured antibody clones from the immune library compared to the naïve library. In the LC of the scFv clones, P residues are only found in immune library scFv clones with an increase of H residues. These findings are similar to previously published data [31,32].

The epitope or antigenic determinant can be divided into two categories, linear and conformational epitopes. Linear epitopes consist of stretches of continuous primary amino acid sequences that are involved in antibody–antigen interactions. On the other hand, conformational epitopes or discontinuous epitopes are made up of amino acids that are scattered at different locations along a plane and brought together in close proximity by protein folding. Therefore, the conformational epitopes on a target protein can only be found in their native state. The scFv clones isolated in this study did not show any binding when the target antigen was in a denatured state, indicating the likelihood of the scFv clones interacting with conformational epitopes. This can only be confirmed either by epitope mapping or using structural bioinformatic approaches [37,38]. However, a competitive ELISA was carried out to determine if the identified scFv clones are binding to the same epitope. The immune library scFv clones did not compete with each other, meaning that both clones are likely to bind to the target antigen at different sites. However, scFv clones obtained from the naïve library exhibited a competitive nature. It is likely that the clones are binding to the same epitope on the antigen. In addition, these clones also compete with clone Ab 20 from the immune library. This means that of the four clones identified, three are likely to bind to the same epitope, with clone Ab 4 being the exception.

Clone Ab 20 and Ab H3R1 have a similar HC V-gene sequence derived from the same VH2 gene family but with different combinations of LC. This may explain the cross-reactivity between Ab H3R1 and Ab 20 as CDR3 of the HC is considered to be a critical factor in determining binding specificities. However, there are two key observations from the data obtained with the scFv clones. Clone Ab 4, although sharing the same V-gene usage as clone Ab 20 and Ab H3R1, did not show any competitive binding. The second is with clone Ab H4R1, which exhibited a different V-gene sequence but binds to a similar epitope to clone Ab H3R1 and Ab 20. This highlights the independence of amino acid residues and gene segment usage for binding preferences. More importantly, it exemplifies the diverse repertoire of the immune system to generate antibodies against similar antigens and epitopes using different gene segment combinations and amino acid propensities.

Not all the residues within the CDR region bind to the antigen [39]. It is estimated that only 20% to 30% of the residues that reside within the CDR regions participate in antigen binding. A CDR on its own may not fold to the same conformation that may be crucial for binding as neighboring residues in the framework regions can dictate the final fold of the CDR. The ability to have multiple clones binding to the same epitope is not unprecedented as there has been a report of an antigen epitope that is recognized by two different antibodies with no sequence similarity [40]. However, from a structural point of view, the structural position of the different clones to the same epitope may differ. The angle at which an antibody binds to the epitope may differ depending on the position of the epitope along the structure of the antigen. This can only be confirmed using structural bioinformatics approaches by analyzing the 3D model of the antibody–antigen interaction through molecular docking simulations [41,42,43].

Titration ELISA was performed to rank the performance of the clones based on their binding abilities. The clone ranked highest in binding ability was clone Ab 4 from the immune library at 10 pg (2.86 × 10^3^ nM), followed by clone Ab H4R1 from the naïve library with binding levels at 100 pg (2.86 nM). This supports the fact that both immune and naïve libraries are able to produce good binders. However, when making a direct comparison between naïve and immune library derived clones, the clones from an immune library generally show higher binding ability against the target antigen due to the affinity-matured repertoire of the immune library. Clones Ab 4 and Ab 20 were used to carry out a pulldown experiment. The choice of clones Ab 4 and Ab 20 was the fact that they bind to different epitopes and are derived from the immune library. Although clone Ab H4R1 from the naïve library showed better binding strength compared to clone Ab 20, the purity of this clone when expressed and purified was not satisfactory. The immunoaffinity pulldown experiment showed the ability of the scFv clones to specifically identify and isolate the target antigen B*m*R1 from total cell lysate with little cross-reactivity to other proteins in the lysate.

This study shows that naïve B cell repertoires also contain receptors that are able to recognize an enormous variety of antigens without any prior exposure. The naïve library used was reported to have a diversity of 10^9^, which is 10 times higher than the diversity of the immune library at 10^8^ [14]. The higher diversity of the naïve library is a general requirement of a good-quality naïve library as the repertoire is generally obtained from B-cells that have not encountered antigen-presentation or further maturation. This is typical of naïve libraries where the main isotype used for repertoire amplification is IgM [14]. However, the ability of the naïve library to generate antibodies against parasitic antigens could be due to the subjective nature of the healthy donors used for the naïve library. This is because it is impossible to select for ‘true’ healthy donors as some may have been exposed to infections earlier in their life without clear clinical symptoms, which could generate the presence of memory cells against the infection. In addition to that, the library generation process allows for combinatorial mixing of antibody genes that may result in combinations that are not present naturally. Therefore, comparative analysis with sera may not represent the same antibody binding patterns that are being presented in the phage library. However, the smaller immune library was sufficient to yield good-quality antibodies against the target antigen. The antibodies produced from an immune library generally exhibit greater affinities compared to antibodies derived from naïve libraries as the immune repertoire would have undergone affinity maturation and is primed after antigen exposure [44]. The ability of the immune library where the repertoire was obtained from chronic infected individuals strengthens the role B cells have in mediating an immune response against filarial infections. Even so, this does not discount the fact that naïve antibody libraries are also useful for antibody generation for infectious agents.

In summary, the present study provides a small glimpse into the complex nature of antibody gene rearrangement for antigen binding. The complex nature and role of gene segment usage and amino acid distribution in CDR of a particular antibody against a target antigen may provide important information for antibody engineering. It is also important to note that the scFv clones identified in this study are potentially useful affinity reagents for biomedical applications, contributing to the control and elimination efforts of lymphatic filariasis in the near future.

## 4. Materials and Methods

### 4.1. Human scFv Library

The construction process for the human scFv immune library for lymphatic filariasis and naïve library was previously described [13,14]. The immune library repertoire was derived from a pool of individuals infected with lymphatic filariasis, whereas the naïve repertoire was obtained from a collection of healthy individuals. The libraries have been applied previously for the isolation of scFv clones against other target antigens [13].

### 4.2. Biopanning

Biopanning was performed using the scFv libraries against the B*m*R1 antigen. The B*m*R1 antigen was expressed and purified according to the published protocol [45]. The biopanning process was carried out as previously reported [4], with slight modifications. The biopanning process was carried out for three rounds instead of four rounds.

### 4.3. Polyclonal and Monoclonal ELISA

Polyclonal and monoclonal phage ELISA was performed as previously reported [4], but with a modification for the naïve library. The modification was 10 μL of 10^12^ M13K07 helper phage (Stratagene, San Diego, CA, USA) was used instead of 10^10^ M13K07 helper phage.

### 4.4. Positive Clone Sequencing

Positive clones were identified through DNA sequencing. Briefly, ELISA positive clones with absorbance reading above 0.5 nm were grown overnight at 37 °C and purified using the Mini prep kit according to the manufacturer’s protocol (Qiagen, Germantown, MD, USA). The purified plasmid DNA was sent for sequencing at First BASE Laboratories Sdn Bhd (Selangor, Malaysia).. The sequencing results were then analyzed using IMGT/VQUEST and VBASE2 [46,47]. The analysis focused on the identification of the V gene, V-gene pairing, CDR length, and amino acid distribution and polarity.

### 4.5. Monoclonal Antibody Protein Expression

Plasmids of positive clones from monoclonal phage ELISA were transformed into SHuffle^®^ Express *Escherichia coli* cells (NEB) for soluble scFv expression [48]. Expression was performed in 500 mL 2-YT broth supplemented with 100 μg/mL ampicillin and 2% glucose. The culture was grown at 37 °C shaking at 200 rpm until the OD_600_ reached 0.6–0.7. The expression culture was induced by adding 1 mM isopropyl-B-d-thiogalactopyranoside (IPTG) and further cultured for 16 h at 25 °C with 180 rpm shaking.

### 4.6. Purification of Monoclonal Antibody Protein

All the scFv proteins were purified using their C-terminal His tag with a nitrilotriacetic acid (Ni-NTA) purification column (Qiagen) according to the manufacturer’s instructions. The resin was washed with distilled water and equilibrated with binding buffer containing 10 mM of imidazole at pH 8.0. The unbound protein contaminants were removed by using the washing buffer containing 10 mM to 40 mM of imidazole. Protein elution was performed using the elution buffer containing 250 mM imidazole and the eluted fractions were analyzed using 10% SDS-PAGE.

### 4.7. Western Blot Analysis

Western blotting of the soluble scFv was carried out using standard protocols. Western blot analysis was performed using HRP conjugated anti-Cmyc antibodies (1:2500) as the secondary antibody. Western blotting for antigen–antibody analysis was performed using non-reducing SDS PAGE. Briefly, the antigen was separated on non-reducing SDS PAGE without β-mercaptoethanol in the sample buffer and heating but in the presence of SDS. The SDS PAGE was carried out at 100 V for 1 h until the front dye reached the bottom of the gel. Then proteins were transferred onto the nitrocellulose membrane using semi dry transfer at 12 V for 30 min. The membrane was then cut into stripes and incubated with the respective scFv clones at 4 °C overnight followed by incubation with HRP conjugated anti-Cmyc antibodies (1:2500). The bands were developed using CN/DAB substrate.

### 4.8. Competitive ELISA

Competitive ELISA was performed similarly, as previously reported [4]. The purpose of this ELISA is to examine the binding site of the scFv clones. Briefly, the plate was coated with 10 μg/well of B*m*R1 antigen. Then the wells were washed, blocked, and incubated with 50 μg/well of purified scFv clone proteins. After 1 h, the wells were washed and 10^5^ of scFv phage was added accordingly and incubated for 1 h RT. The wells were then washed and incubated with 100 μL HRP conjugated anti-M13 antibody (1:5000 dilution) for 1 h at RT. Then the plate was washed and 150 μL ABTS substrate was added. The absorbance value was measured at 405 nm using a SkanlT absorbance reader (Thermo Scientific, St Peters, MO, USA) after 30 min incubation in the dark. The absorbance values from the control wells were subtracted from test wells to obtain total absorbance.

### 4.9. Antibody Titration ELISA

Antibody titration ELISA was performed to determine the limit of binding of the identified anti-B*m*R1 scFv clones. In total, four soluble scFv clones were analyzed, two from the immune library (Ab 4, Ab 20) and two from the naïve library (Ab H4R1 and Ab G1R1). Briefly, ELISA was performed by coating 10 μg/well of B*m*R1 antigen on Maxisorb ELISA microtiter plate (Nunc, New York, NY, USA) with 1XPBS, pH 7.4 overnight. The coated wells were washed with PBST (PBS with 0.5% Tween 20) and blocked with MPBST (2% skim milk in PBST) for 1 h at room temperature (RT). After washing, each well was incubated with the scFv protein at concentrations ranging from 100 μg/well to 100 fg/well for 2 h at RT. The plate was then washed and incubated with 150 μL HRP conjugated anti-Cmyc secondary antibody diluted 1:5000 in MPBST for 1 h at RT. The plate was washed three times with PBST and 150 μL of ABTS substrate was added and left to incubate in the dark for 30 min. The absorbance value was read at 405 nm using the SkaniT absorbance reader (Thermo Scientific).

### 4.10. Immunoaffinity Pulldown

Selected scFv clones were coupled to cyanogen bromide (CNBr)-activated sepharose resin based on previously published protocols [49]. The coupling efficiency was calculated by performing SDS PAGE analysis and protein concentration using protein supernatants of before and after coupling. Then, the scFv-coupled resin was packed into individual columns. The column binding efficiency was evaluated using crude protein and analyzed using SDS PAGE.

## Abbreviations

PCR	Polymerase chain reaction
Ig	Immunoglobulin
scFv	Single-chain variable fragments
mAb	Monoclonal antibody
CDR	Complementary determining region
HC	Heavy chain
LC	Light chain
CNBr	Cyanogen bromide
